# Multicentre Pilot Study to Evaluate the Efficacy of Targeted Exercise in Combination with Cytisinicline on Smoking Cessation at 12 Months: MEDSEC-CTA

**DOI:** 10.3390/healthcare12242516

**Published:** 2024-12-12

**Authors:** Sofia Ruiz-Salcedo, Antonio Ranchal-Sanchez, Javier Ruiz-Moruno, Jaime Montserrat-Villatoro, Jose Manuel Jurado-Castro, Esperanza Romero-Rodriguez

**Affiliations:** 1 Maimonides Institute for Biomedical Research of Córdoba (IMIBIC), Reina Sofía University Hospital, Córdoba University, 14004 Córdoba, Spain; sofiaruizsalcedo15@gmail.com (S.R.-S.); jruiz.moruno@telefonica.net (J.R.-M.); jaime.monserrat.sspa@juntadeandalucia.es (J.M.-V.); emromerorodriguez@gmail.com (E.R.-R.); 2 Córdoba and Guadalquivir Health District, 14011 Córdoba, Spain; 3 Department of Nursing, Pharmacology and Physiotherapy, Faculty of Medicine and Nursing, University of Cordoba, 14004 Córdoba, Spain; 4 Centro de Investigación Biomédica en Red de Fisiopatología de la Obesidad y Nutrición (CIBEROBN), Instituto de Salud Carlos III (ISCIII), 28222 Madrid, Spain; 5 Ciencias De La Actividad Física y El Deporte, Escuela Universitaria de Osuna (Centro Adscrito a la Universidad de Sevilla), 41640 Osuna, Spain

**Keywords:** tobacco use cessation, cytisine, exercise, randomised controlled trials as topic

## Abstract

**Background/Objectives:** Current scientific evidence shows both the relationship between good physical condition and a lower incidence of certain chronic diseases (including smoking), as well as the efficacy of cytisinicline. The aim of this protocol is to evaluate the efficacy of the synergistic effect of the combination of targeted physical exercise, together with brief advice and taking the drug cytisinicline, to achieve smoking cessation. **Methods**: We propose an experimental, multicentre, randomised, controlled study with two parallel arms to be carried out by a multidisciplinary team in the primary care setting of the Andalusian public health system (APHS) in Spain, with a follow-up of 12 months. The population sample will include people who are aged between 18 and 65 years and meet the criteria to be eligible for treatment with cytisinicline financed by the APHS: smokers of 10 or more cigarettes per day who are in the determination/action phase for smoking cessation of the transtheoretical model of Prochaska and Diclemente, willing to start anti-smoking treatment imminently and confirmed, with high dependence to nicotine (Fagerström’s test ≥ 7), with a motivation to quit smoking according to the Richmond test (≥6), and who have made a previous smoking cessation attempt in the last year. The study consists of two treatment arms. EXPERIMENTAL ARM: Smokers who are going to be prescribed targeted physical exercise and brief advice to stop smoking while receiving cytisinicline treatment (1.5 mg tablets) according to the care process. CONTROL ARM: Smokers will receive a standard leaflet explaining physical exercise to the general population and brief advice on smoking cessation when starting cytisinicline treatment, according to the care process. The calculated sample size is 75 participants per arm. **Results:** The primary results will allow us to estimate the efficacy of prescribing physical exercise as an adjuvant therapy to classic multicomponent treatment, incorporating it as an additional element to be considered when it is accepted by the smoker. **Conclusions:** This protocol (NCT06579846) evaluates the efficacy of combining physical exercise, brief advice, and cytisinicline to support smoking cessation, improve fitness, and reduce smoking-related comorbidities

## 1. Introduction

Increasing importance is being given to the so-called “physical exercise prescription”. Proof of this is the recent specific plan implemented in Andalusia, Spain [1]. Current scientific evidence shows the relationship between good physical fitness and reduced incidence of certain chronic diseases, ranging from “some evidence of reduced disease rates in the case of lung disease (with few studies conducted)” to “good evidence” for hypertension and “excellent evidence” for reduced disease rates in obesity, coronary heart disease and all-cause mortality [2,3].

Smoking can be considered a chronic, addictive disease with a tendency to relapse. It has associated comorbidity, such as multiple cancerous processes, chronic obstructive pulmonary disease, and other pathologies that also constitute important cardiovascular risk factors, such as hypertension and obesity [4,5]. In this respect, obesity is a common barrier that smokers, especially women, often cite as a reason for delaying quit attempts. It is therefore interesting to consider adjuvant therapy that also helps to control risk factors, such as the prescription of physical exercise, in people who decide to quit smoking. This may also be useful for dealing with the withdrawal syndrome caused by smoking cessation. In this regard, a meta-analysis published in 2023 indicates that short interventions with physical exercise are effective in reducing craving and withdrawal symptoms in smokers, as well as helping to improve their mood [6]. In the same vein, a clinical trial to test the efficacy of a 12-week exercise intervention in conjunction with health education interventions also shows greater reductions in craving while attenuating the negative emotional effect that is generally associated with craving [7]. Cytisinicline is a recently financed drug by the Spanish Health System to help quit smoking, increasing the demand to cope with this addiction [8,9,10]. In this sense, the aim of this protocol is to evaluate the efficacy of the synergistic effect of the combination of targeted physical exercise, together with brief advice and taking the drug cytisinicline, to achieve smoking cessation.

## 2. Materials and Methods

### 2.1. Study Design

A multicentre, randomised and controlled experimental study with two parallel branches, to be carried out by a multidisciplinary team of different specialties (medicine, pharmacology and physical education sciences) of the Maimonides Institute of Biomedical Research of Cordoba (IMIBIC), in the field of the Andalusian Public Health System (APHS), is proposed.

### 2.2. Study Population

The study population will be made up of people who smoke and who are assigned to different health centres in the Health District of Cordoba and Guadalquivir, belonging to the APHS. This Health District has a total population of 437,883 users. The health centres to be included in the study are: “Aeropuerto” (15,496 users), “Carlos Castilla del Pino” (serving 21,533 users), and “Bujalance” (4537 users), including patients between 18 and 65 years old [11]. The study population will be composed of individuals aged 18 to 65, drawn from the 19.7% of the Andalusian population who smoke [12].

### 2.3. Sample Size

In order to calculate the sample size, we have taken into account the results obtained in four experimental studies very similar in objective, intervention and time of evolution to ours [13,14,15,16]. One [13] was discarded because it did not have results 12 months after the start of the intervention.

An aggregation of results weighted by sample size yielded a difference between the control and experimental groups of 3.18% smoking cessation at 12 months. For a delta of 5%, an alpha of 5% in a hypothesis of superiority, and a beta error of 20%, a sample size of 289 participants per branch was obtained. As it was not feasible to recruit such a large number of patients, we set a target of 75 patients per arm (150 in total), performing a sensitivity analysis, if no differences were found, to check if they were not found for reasons related to the sample size.

### 2.4. Sample Selection or Eligibility Criteria

#### 2.4.1. Inclusion Criteria

The sample will include people who, agreeing to participate in the study, are aged between 18 and 65 years and meet the criteria to be eligible for treatment with cytisinicline financed by the APHS. These criteria are smokers of 10 or more cigarettes per day, who are in the determination/action phase for smoking cessation of the transtheoretical model of Prochaska and Diclemente [10], willing to start anti-smoking treatment imminently and confirmed, with a high dependence on nicotine (Fagerström’s test ≥ 7), with a motivation to quit smoking according to the Richmond test (≥6), who have made a previous smoking cessation attempt in the last year, and who are covered by the APHS.

#### 2.4.2. Exclusion Criteria

The following will be causes for exclusion in this study: persons suffering from pathologies that prevent physical exercise (malignant hypertension, heart failure, hyperthyroidism, peripheral arterial disease), or other diseases that, in the opinion of the research team, contraindicate the practice of physical exercise or their participation in the study, either due to a foreseeable lack of adherence or risk of adverse events; changes in the usual treatment in the last 90 days; and inability to sign informed consent. Also, suffering from pathological processes that limit life expectancy significantly (<5 years), and the existence of contraindications established in the technical data sheet of cytisinicline [17]: pregnancy, breastfeeding, hypersensitivity to cytisinicline, unstable angina, history of recent myocardial infarction, clinically relevant arrhythmias, recent history of stroke.

### 2.5. Recruitment of Participating Smokers

Recruitment will be carried out by Family Medicine physicians in the Primary Care clinics of the aforementioned health centres of the APHS (Aeropuerto, Bujalance and Castilla del Pino). The Family Medicine physicians will be responsible for evaluating and confirming the medical conditions of the study population, based on medical records and their medical criteria.

The patient will be proposed to participate in the study in routine consultations when he/she has attended due to a desire to stop smoking and is going to receive pharmacological treatment with cytisinicline. Nursing staff from each of the health centres may participate in the recruitment process by virtue of the Resolution of 22 March 2024 of the Directorate General for Public Health and Equity in Health, which validates the “Guide for the indication, use and authorisation of dispensing of medicines subject to medical prescription by nurses: smoking cessation” [18].

The recruitment period will last 6 months, with a mid-term review after 6 months and a final review after 12 months.

### 2.6. Branch Allocation and Randomisation

To prevent some of the variables from influencing smoking cessation, adaptive randomisation will be used. This is a technique used in clinical trials that allows treatment allocation to participants to be changed over the course of the study based on the cumulative results. Unlike traditional randomisation, where treatment assignment is fixed and decided before the study starts, adaptive randomisation adjusts the probability of treatment assignment on the fly, which may increase the efficiency of the study and improve the likelihood of detecting significant differences between treatments. The first 20 patients will be randomised in a simple way, using a randomisation table. From patient 21 onwards, the composition of each of the groups will be taken into account to weigh the probabilities of falling into one group or the other.

### 2.7. Source of Data

Data will be obtained by clinical interview with the participants and, if allowed, from the patient’s digital medical history in DIRAYA (the APHS electronic medical record).

### 2.8. Data Management

The data will be collected and managed using the electronic data capture tool REDCap (Research Electronic Data Capture), a platform used in the field of independent public research, hosted on a virtual server located at the Reina Sofia University Hospital (Cordoba, Spain). This server is managed by the same technical team that manages the health data of patients at the hospital. REDCap (https://projectredcap.org/, accessed on 10 December 2024) is a secure, web-based software platform designed to support data capture for research studies, providing: (1) an intuitive interface for validated data capture; (2) audit trails for tracking data manipulation and export procedures; (3) automated export procedures for seamless data downloads to common statistical packages; and (4) procedures for data integration and interoperability with external sources.

Access to this platform will only be allowed to the researchers of this study, preventing these data from being accessed by third parties.

### 2.9. Study Variables

The data collected will correspond to the following study variables, both outcome and explanatory.

Outcome variables will be: number of cigarettes smoked daily (discrete quantitative) *; smoking cessation (dichotomous): variable calculated on the basis of whether the number of cigarettes is equal to 0, and exhaled air CO-oximetry detects a value equal to or less than 10 ppm *; blood pressure (continuous quantitative); heart rate (continuous quantitative); weight in kilograms (continuous quantitative) *; and Body Mass Index (BMI) (continuous quantitative): variable calculated on the basis of weight and height) *.

Explanatory variables are sex (qualitative polychotomous); age (discrete quantitative); occupation (qualitative); postcode of residence (qualitative); cumulative smoking (discrete quantitative) *; and physical nicotine dependence measured by the Fagerström test [19] (discrete quantitative); psychological dependence, as measured by Glover Nilsson’s test [20] (discrete quantitative); motivation to quit smoking, as measured by the Richmond test [21] (discrete quantitative); weekly alcohol consumption (Standard Drinking Units-UBE) (discrete quantitative) *; World Health Organisation (WHO) Global Physical Activity Questionnaire (GPAQ) [22] and Benisovich’s physical activity self-efficacy scale [23].

(*) Some of the outcome variables can be considered intermediate variables of other explanatory variables, due to their causal relationship.

### 2.10. Material Used for Smoking Determination and Other Instruments

A CO-oximeter of the brand “Co Check”, sold by MD Diagnostics Ltd. (Kent, UK), distributed by Sibel S.A. (Barcelona, Spain), code 01PLS18B, with a sensitivity of 1 ppm and an accuracy (repeatability of 2%, purchased in 2023) will be used to measure smoking and smoking cessation. To differentiate between smokers and ex-smokers, the criteria established in the Andalusian Comprehensive Smoking Plan (ACSP) will be followed [24].

Regarding arterial pressure, an OMROM M6-Confort (HEM-7000-E-V) (Omron HealthCare, Kyoto, Japan) will be used for the blood pressure measurement. The height and weight (Barys Plus Ref ASI06472; Asimed, Sibel group, Barcelona, Spain) of the participants will be recorded.

### 2.11. Intervention

Interventions will be developed by a multidisciplinary team with experience in tackling tobacco consumption. Physical exercise recommendations will be made by specialists in Medicine, Physical Education, and Sport Sciences and will be carried out in line with the Andalusian Physical Exercise Prescription Plan (APEPP) [1], the recently published regional plan for Andalusia. Therefore, this study consists of two treatment arms:

A. Smokers who are going to be prescribed targeted physical exercise and brief advice to stop smoking while receiving cytisinicline treatment (1.5 mg tablets) according to the care process: EXPERIMENTAL ARM.
B. Smokers who will receive a standard leaflet explaining physical exercise to the general population and brief advice on smoking cessation when starting cytisinicline treatment, according to the care process: CONTROL ARM.

#### 2.11.1. Prescription of Targeted Physical Exercise

The baseline level of physical activity and physical exercise will be determined using the modified version of the Global Physical Activity Questionnaire (GPAQ) [22] which will be administered to participants in the experimental arm. In this regard, study participants will be classified according to low (inactive), moderate, and high levels of physical activity following the criteria of the GPAQ [22].

For the prescription of targeted physical exercise, the guidelines established by the American College of Sports Medicine (ACSM) [2], those indicated in the “Guide for professionals” of physical activity recommendations and prescription of physical exercise for health” recently published by the Andalusian Regional Government [25], and those suggested in the Stockton review [16] will be followed.

A combined aerobic and strength exercise, and Flexibility or Range of Motion and Neuromotor Capabilities protocol will be implemented, designed to promote adherence and supervised through continuous monitoring. Participants will engage in moderate-intensity aerobic exercise for a minimum of 30 min on alternate days, complemented by strength exercises targeting the major muscle groups. Exercise intensity will be monitored using beats per minute, either manually or by means of smartwatch-type devices in line with the “ACTIVITAL” platform recently implemented by the Andalusian Regional Government [26].

A standard leaflet with basic physical exercise recommendations will be provided at the start of the prescription (Table 1, Table 2 and Table 3). In addition, inactivity or physical activity habits (such as walking to work), as well as regular physical exercise and sports activities will be collected at the initial interview in order to include the smoker in the appropriate arm of the study.

To achieve individualisation and progression, a multi-level system (low, moderate, and high) has been implemented (Table 1, Table 2 and Table 3). Participants will be assigned to an initial level based on their GPAQ results and individualised follow-up assessments at each visit. This system allows progression to higher levels as their physical condition improves. For example, a participant may progress from low to moderate level over time, but this advancement may vary across exercise categories. For instance, while cardiorespiratory exercises may progress to a higher level, strength exercises might remain at the initial level.

To clarify this structure, exercise types within each level have been specifically labelled (e.g., Low Level 1.1 for Cardiorespiratory Endurance, Low Level 1.2 for Muscle Strength and Endurance, etc.) and are detailed in (Table 1, Table 2 and Table 3).

The prescription includes explicit descriptions of the exercises, intensities, and progression criteria, ensuring replicability. The load progression will follow predefined criteria, considering physical responses, including improvements in the number of repetitions, sets, session durations, and intensity levels (e.g., %HR max or %1RM). Adjustments will be made based on participant feedback and performance, ensuring gradual and safe increases in load. The physical exercise prescribed shall be individualised and adapted to the person’s physical condition, motivating and facilitating social relations and non-detrimental. The principles of progression and alternation shall also be respected. The exercise will be progressive depending on the volume and intensity applied to each person according to their physical condition and supervised by experts in the field. If a participant has an injury or discomfort that prevents them from performing any of the proposed exercises, an alternative will be offered to achieve the same type of work.

Directed physical exercise will be recorded in an Electronic Data Collection Notebook (CRDe) and in the digital medical record using DIRAYA. To encourage adherence, a continuous, standardised and motivating follow-up will be carried out by video call or telephone call. Only study investigators will have access to this CRDe. In addition, this system has a mechanism of roles and profiles that allows each researcher to be assigned what information they can access, so it can be individualised.

#### 2.11.2. Brief Advice

The brief advice will be consensual and provided by Family and Community Medicine specialists. It will be carried out in line with the guidelines established in the comprehensive plan against smoking in Andalusia, the integral smoking plan (PITA) [24]. Advice should be brief, opportunistic, personalised and clear (recommendation A). The strategy of this advice follows the mnemonic rule of the “5 As” (in Spanish): find out, advise, appreciate, help, and agree, the appropriate follow-up plan depending on the assigned arm, taking into account that the smokers participating in this study should be in the determination-action phase of the stages of change [10].

#### 2.11.3. Pharmacological Treatment with Cytisinicline

Treatment with cytisinicline of 1.5 mg tablets will be carried out by APHS physicians in order to meet the criteria established for its prescription, with clinical variables being recorded in the DIRAYA computer programme. In Spain, the cytisinicline manufacturers are Aflofarm (Pabianice, Poland) and Adamed (Pieńków, Poland), who indicate in the technical sheet that the patient should avoid smoking no later than the fifth day of treatment. In case of failure, it must be discontinued and may be resumed after two months. According to the datasheet, the duration of treatment (ingested) is twenty-five days. The treatment regimen [17] is shown in Table 4.

### 2.12. Participant Chronology and Schedule of Visits

**Figure 1:**
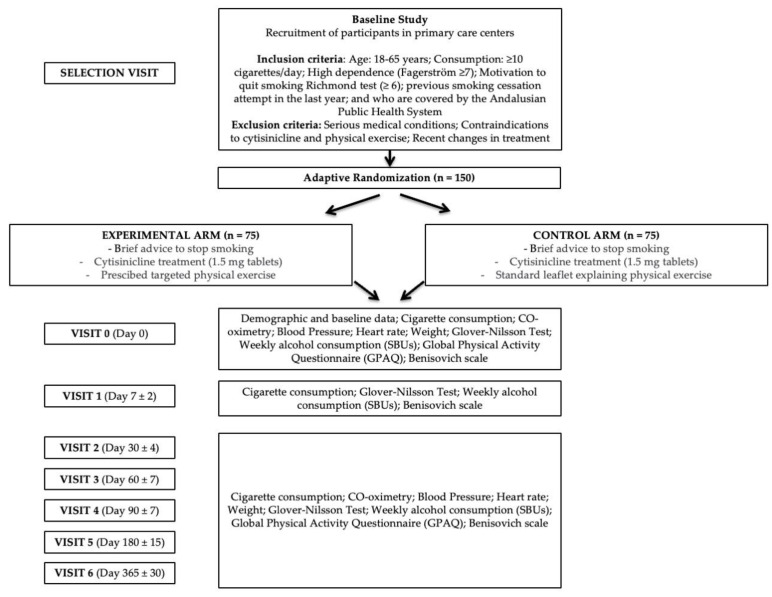
Flowchart study protocol.

All participating smokers will be followed up, either in person or by telephone, according to the plan established in this study and at the visits indicated below (Figure 1):

- Selection visit (Day 0): FACE-TO-FACE. The potential participant is proposed to participate in the study. A check is made to ensure that the patient has no medical or other history that might contraindicate the prescription of physical exercise. The patient receives the information and signs the informed consent form if he/she wishes to be included in the study.
- Visit 0 (Day 0): FACE-TO-FACE. Baseline data are collected on the previously mentioned variables. The necessary criteria for prescription are completed. The drug is prescribed to be approved within 24h, and a treatment start date is set.
- Visit 1 (Day 7 ± 2): TELEPHONE. Data are collected, and the participant is asked if there are any problems with the motivation to continue.
- Visit 2 (Day 30 ± 4): FACE-TO-FACE. Data are collected, and questions are asked if there have been any problems with the physical exercise, motivating for its continuation.
- Visit 3 (Day 60 ± 7): FACE-TO-FACE. Data are collected, and questions are asked if there have been any problems with the physical exercise, motivating for its continuation.
- Visit 4 (Day 90 ± 7): FACE-TO-FACE. Data are collected, and a question is asked as to whether there have been any problems with the physical exercise, motivating the patient to continue.
- Visit 5 (Day 180 ± 15): FACE-TO-FACE. Data are collected, and a question is asked as to whether there have been any problems with the physical exercise, motivating the patient to continue.
- Visit 6 (Day 365 ± 30): FACE-TO-FACE. Data are collected.

### 2.13. Statistical Analysis Plan

For the statistical analysis, the R programming language and its IDE (Integrated Development Environment) will be used: RStudio, in its most updated version.

Initially, a descriptive analysis of the baseline characteristics of the study population will be carried out using median and interquartile ranges for quantitative variables and absolute and relative frequencies for categorical variables.

To compare groups by bivariate analysis, Wilcoxon, Chi-square or Fisher tests will be used, as appropriate. Causal inference between intervention and outcomes will be made through multivariate analysis using logistic regression for the dichotomous variable “Smoking cessation” and linear regression for the variable “Number of cigarettes”, adjusting for potential confounders.

All analyses shall be conducted on an intention-to-treat basis, and no missing data shall be imputed.

Statistical significance will be established at a *p*-value < 0.05. Further analyses, including sensitivity and subgroup analyses, will be conducted to explore the robustness and generality of the findings.

### 2.14. Bias Control

Selection bias will be controlled by applying the same selection criteria to the participants in the different groups to be compared, which will have identical monitoring. In addition, in the collection of information, a record of losses to follow-up will be kept, collecting as much information as possible about them. Regarding misclassification bias, smoking will be considered as a criterion of truth, the measurement of 6 or more ppm of CO in exhaled air by CO-oximetry. Finally, multivariate analysis using logistic regression will control for various confounding factors.

### 2.15. Security Event Reporting

As this is a NON-PHARMACOLOGICAL intervention study, adverse events related to the drug used as inclusion criteria will be reported according to standard practice protocol through the Spontaneous Reporting of Adverse Reactions Programme of the Andalusian Centre for Pharmacovigilance.

### 2.16. Ethical and Regulatory Framework

This study will be conducted in strict accordance with the ethical principles for research involving human participants, in accordance with the Declaration of Helsinki-Fortaleza, and with the legal regulations in force on research and personal data protection (Law 14/2007 on Biomedical Research and Organic Law 3/2018 on Personal Data Protection and guarantee of digital rights), Regulation (EU) 2016/679 of the European Parliament and of the Council, of 27 April 2016 on the protection of natural persons with regard to the processing of personal data and on the free movement of such data and repealing Directive 95/46/EC (General Data Protection Regulation), Organic Law 3/2018 of 5 December on the Protection of Personal Data and the guarantee of digital rights, and Law 14/2007 of 3 July on Biomedical Research.

This study is committed to respecting the rights of participants, including their dignity, autonomy (Law 41/2002, basic law regulating patient autonomy and rights and obligations regarding clinical information and documentation) and well-being, and to conducting the research in a way that maximises the potential benefits to society while keeping risks and discomfort to participants to a minimum.

### 2.17. Ethics Committee

Before starting the study, the protocol was reviewed and approved by the Cordoba Research Ethics Committee by resolution of 30 July 2024 with code SICEIA-2024-001451.

### 2.18. Amendments to the Protocol

Any amendment to be made by the research team must be submitted to and obtain prior authorisation from the competent ethics committee for each amendment, which, failing this, will be the Cordoba Research Ethics Committee.

### 2.19. Informed Consent

All participants will be informed of the objectives, procedures, potential benefits and risks associated with the study, as well as their right to withdraw from the study at any time without any consequences for their medical care. Written informed consent will be obtained from each participant prior to inclusion in the study.

### 2.20. Confidentiality and Data Access Policy

The confidentiality of participants’ personal data will be guaranteed at all times, and such data will be processed in accordance with the applicable regulations on personal data protection, ensuring their anonymity and the security of their storage and processing. Only the research team will have access to identifiable data.

### 2.21. Declaration of Conflict of Interest

The members of the research team declare that they have no conflicts of interest in carrying out this study.

### 2.22. Performance Advertising Policy

Any disclosure of results shall be made in such a way that it is not possible to identify any participant.

The results of this study, whatever they may be, will be published in indexed scientific journals and subsequently disseminated through digital media (social networks, websites) to reach the general population.

### 2.23. Gender Perspective

A gender perspective in research is a necessary approach to properly understand and address the differences and inequalities that exist between men and women in relation to tobacco use. The inclusion of this perspective not only enriches the analysis of data but also improves the effectiveness of health interventions and policies aimed at reducing smoking and its consequences.

Numerous studies have shown that there are significant differences in smoking patterns between men and women. Historically, men have had higher rates of tobacco use than women. However, the gap has been narrowing in recent decades in many regions of the world [27]. In addition, the motivations and circumstances that lead individuals to initiate and maintain tobacco use can vary considerably by gender. For example, women may be more influenced by factors related to stress and body image, while men may be more influenced by social and cultural factors [28,29]. In this sense, our study contemplates a descriptive analysis of the data for each group, including gender as a qualitative explanatory variable. In addition, we address a common barrier to smoking among women smokers, namely weight gain after smoking cessation.

The impact of tobacco use on health also shows gender differences. Female smokers have a higher risk of developing cardiovascular disease compared to male smokers and also face unique risks, such as complications during pregnancy and negative effects on reproductive health. Furthermore, certain types of tobacco-related cancers, such as lung cancer, may manifest differently in men and women, underscoring the need for specific approaches to prevention, diagnosis and treatment [30,31].

Tobacco control interventions and policies may have different effectiveness according to gender. Smoking cessation programmes that do not consider gender differences may be less effective for one group or the other. For example, women may respond better to interventions that address weight and stress management, while men may benefit more from approaches that emphasise the economic and social impact of smoking. Thus, a gender perspective allows for the design of more tailored and effective interventions [32]. In this regard, our study considers two different smoking cessation interventions, both of which have an impact on aspects such as weight gain (physical exercise) or economic impact (NHS-funded medication).

Gender inequalities may also influence access to and adherence to the resources needed to quit smoking. Women may face additional barriers, such as lower income, caregiving responsibilities and social stigma, which make it difficult for them to access smoking cessation programmes [33]. As mentioned above, one way to address these barriers is the use of a drug that is currently funded by the NHS, which facilitates access for people with fewer financial resources. On the other hand, the prescription of targeted physical exercise is intended to ensure a close follow-up of patients, which would encourage adherence to these programmes among this group of smokers. Integrating a gender perspective in this type of research contributes to a more inclusive science and the promotion of public health in an equitable manner.

### 2.24. Registration in Clinical Trials

The study is registered in clinical trials under the code NCT06579846 and can be consulted at the following link:

https://clinicaltrials.gov/study/NCT06579846?viewType=Card&sort=StudyFirstPostDate&limit=50&term=cytisinicline&intr=Exercise&rank=1 (accessed on 10 December 2024).

In this respect, the research protocol complies with the SPIRIT (Standard Protocol Items: Recommendations for Interventional Trials) guidelines for the design and reporting of clinical trials.

## 3. Expected Results

The primary results will allow us to estimate the efficacy of prescribing physical exercise as an adjuvant therapy to classic multicomponent treatment, incorporating it as an additional element to be considered when it is accepted by the smoker. Secondly, we will find out, in young smokers, its acceptance as an optional therapy, with the high cost-effectiveness of quitting smoking as early as possible in the life of smokers.

We will also learn about the influence of the prescription of physical exercise in the female gender in relation to barriers such as weight gain and/or obesity, generally associated with smoking cessation. In addition to the potential added benefit in the control of associated dependent variables such as arterial hypertension, hyperglycaemia and dyslipidaemia, which are directly related to “physical inactivity” as an independent variable that is becoming increasingly relevant in our digital society.

The results obtained would be immediate, in terms of improving the smoker’s health, from the moment the smoking cessation process begins in synergy with the prescription of controlled physical exercise. We believe, therefore, that the results will affect the improvement in the level of health of smokers who achieve abstinence from smoking by improving their quality of life and, in the long term, their average life expectancy through the control of at least two risk factors such as smoking and physical inactivity. And not only to facilitate immediate smoking cessation but also to prevent craving and/or subsequent relapses, with a positive impact on the quality of life of people who achieve smoking cessation.

With regard to the improvement in public health, we understand that the results will favour the reduction of the prevalence of active smoking. Also, passive smoking in those people who involuntarily breathe in smoke from cigarette combustion when living with smokers in the study population. Moreover, reducing smoking has a positive impact on environmental health, in line with the Sustainable Development Goals (SDGs) and the 2030 Agenda of the UN General Assembly. In this sense, the results could be framed along the lines of “health and well-being”, “climate action” (as it implies improving the environment by reducing waste in the form of cigarette butts and environmental tobacco smoke), dangers such as fires from incandescent cigarette butts, and “reducing inequalities” since the most vulnerable people smoke, and more intensively.

## 4. Discussion

To date, there are no studies comparing the efficacy of prescribing physical exercise as an adjunctive treatment to cytisinicline and brief advice to achieve smoking cessation, which is the aim of this research protocol.

Physical exercise per se can activate the dopamine reward system, facilitating the reduction of craving and withdrawal symptoms [34]. Recent studies show a relationship between positive and negative effects and craving. Kunicki et al. [7] suggest that a negative effect is associated with craving, which can be mitigated by exercise, finding greater reductions in craving in smokers who engaged in physical exercise. However, Stockton et al. indicate in their clinical trial that physical exercise does not improve smoking cessation, compared to so-called “wellness counselling”, although they draw attention to the importance of “individualisation” [16].

Regarding the role of physical exercise as an adjunctive treatment, some authors have investigated the synergy with medication against smoking. Thus, regarding the efficacy effects of physical exercise programmes combined with nicotine replacement therapy (NRT), Chen et al. [34] show in their systematic review of clinical trials moderate-high quality evidence that physical exercise can help NRT in short-term smoking cessation [24], suggesting future trials that include larger sample sizes, as well as strategies to increase adherence to exercise in the long term.

We know that NRT is one of the smoking cessation therapies that use classic so-called “first-line” drugs, the other first-line drugs being varenicline and bupropion. Both cytisinicline and varenicline are drugs specifically designed to aid smoking cessation, although the latter has been temporarily withdrawn from the market in Spain, unlike bupropion, which was initially designed as an antidepressant drug. The literature reviewed includes cytisinicline among the second-line smoking cessation drugs.

Cytisinicline is currently funded by the Spanish National Health System (SNHS). In this respect, the 2022 Programme of Preventive Activities for Health (PPAH) states that “payment coverage of smoking cessation treatment that is comprehensive, barrier-free and widely promoted increases the use of these services and leads to higher rates of successful quitting” [35]. The evidence on the efficacy of this drug is so far controversial because, although it was already used in Eastern European countries, it was the West who, in 2011, conducted the first clinical trial against a placebo with favourable results [36]. Subsequently, a systematic review and meta-analysis study on the efficacy of cytisinicline, conducted in 2013, also endorsed its efficacy [37]. Subsequent clinical trials comparing it with nicotine [38] and with varenicline [39,40] support its efficacy, with the ORCA-1 trial being the most powerful study to date in adult smokers [41].

In Spain, where smoking prevalence remains high, with a proportional increase in women [4,42], some studies have already provided information on the satisfaction and tolerability of cytisinicline [43] but not on its efficacy in synergy with controlled physical exercise. There is also a lack of studies on combined interventions involving young smokers, especially after the recent pandemic, due to its harmful effects on undergraduate students [44]. In this sense, intervention by means of “exercise prescription”, together with treatment with brief advice and cytisinicline, has the added purpose of combating weight gain, inactivity, and comorbidities associated with smoking (hypertension, diabetes, etc.), potentially favouring adherence to multicomponent treatment, in addition to smoking abstinence and tackling the barrier of weight gain, which is of particular concern to women smokers as mentioned above.

To implement it, the ideal level for treating smoking is the primary care centres. The reasons for this argument are both because they are the first level of access to the SNHS for smokers and because of the continuity and follow-up they allow during the process of abstinence from smoking. Health centres are also the first level for the initial prescription of physical exercise and its follow-up. This is, therefore, an opportunity to introduce other variables in the digital medical record, such as physical inactivity and the prescription of controlled or directed physical exercise.

### Limitations of the Project

This project should be seen in the context of its limitations. Firstly, the sample size could be an issue. However, the sample selected is representative of the study population, which is determined by the sample size, randomisation and selection criteria. Secondly, there is the possibility of losses during the course of the project, which is intended to be reduced by conducting an intention-to-treat analysis and by conducting follow-ups by telephone.

On the other hand, the project has the strengths of being a feasible, innovative and relevant study in terms of health gains, life expectancy, and the benefits in quality of life and well-being for people who meet the proposed objective by carrying out a programme that takes into account the quantity and quality of physical exercise performed [2]. We believe that the results obtained will be transferable to other countries where the same drug is dispensed and that the long-term results will be beneficial.

## 5. Conclusions

In conclusion, this study protocol (NCT06579846) aims to evaluate the efficacy of the synergistic effect of the combination of prescribed targeted physical exercise as an adjuvant therapy, together with brief advice and taking the drug cytisinicline to achieve smoking cessation in 18 to 65 years old smokers, through a multicentre, randomised and controlled experimental study with two parallel branches, to be performed by a multidisciplinary team, in primary care. The protocol provides specific interventions to achieve individualisation and progression, a multi-level system (low, moderate, and high) that could favour good physical fitness as well as reduced incidence of inactivity and certain chronic smoking-associated comorbidity, such as multiple cancerous processes, chronic obstructive pulmonary disease, and other pathologies that also constitute important cardiovascular risk factors, likes hypertension, weight gain and obesity, which are usually a common barrier to quit smoking, especially for women.

## Figures and Tables

**Table 1:** Low-level physical exercise program: activities, frequency, intensity, and progression.

Visit	Level Exercise	Type of Exercise	Proposed Exercise (Sets and Repetitions)	Frequency (Days/Week)	Intensity (~%HR max/~% 1RM)	Duration per Type (min)
Visit 0 (Day 0)	Low-Level 1.1	Cardiorespiratory Endurance	Slow-paced walking or stationary bike	3–4	Low (~55–60% max HR)	20
	Low-Level 1.2	Muscle Strength and Endurance	Bodyweight squats (3 × 10), knee push-ups (3 × 8), lunges (3 × 8), heel raises (3 × 10), plank (3 × 10 s)	2–3	Bodyweight exercise or Low (~30–50% 1RM)	20
	Low-Levell 1.3	Flexibility or Range of Motion	Basic stretches (15 × 30 s, 4 repetitions per muscle group)	2–3	Low	5
	Low-Level 1.4	Neuromotor Capabilities	Straight-line walking	2–3	Low	
Visit 2 (Day 30 ± 4)	Low-Level 2.1	Cardiorespiratory Endurance	Brisk walking or moderate stationary bike	3–4	Moderate (~65–75% max HR)	25
	Low-Level 2.2	Muscle Strength and Endurance	Bodyweight squats (3 × 12), inclined push-ups (3 × 8), lunges (3 × 10), heel raises (3 × 12), plank (3 × 15 s)	3	Bodyweight exercise or low (~30–50% 1RM)	20
	Low-Level 2.3	Flexibility or Range of Motion	Basic stretches for legs and trunk	2–3	Low	5
	Low-Level 2.4	Neuromotor Capabilities	Static balance exercise (standing on one leg)	2–3	Low	
Visit 3 (Day 60 ± 7)	Low-Level 3.1	Cardiorespiratory Endurance	Brisk walking or steady pace cycling	3–4	Moderate (~65–75% max HR)	30
	Low-Level 3.2	Muscle Strength and Endurance	Bodyweight squats (3 × 15), wall push-ups (3 × 10), lunges (3 × 12), heel raises (3 × 15), plank (3 × 20 sec)	2–3	Bodyweight exercise or moderate (~50–70% 1RM)	25
	Low-Level 3.3	Flexibility or Range of Motion	Basic stretches for shoulders and hips	2–3	Low	5
	Low-Level 3.4	Neuromotor Capabilities	Slow lateral movements	2–3	Low	
Visit 4 (Day 90 ± 7)	Low-Level 4.1	Cardiorespiratory Endurance	Brisk walking or light stationary cycling	3–4	Moderate (~65–75% max HR)	30
	Low-Level 4.2	Muscle Strength and Endurance	Knee push-ups (3 × 12), bodyweight squats (3 × 15), lunges (3 × 15), heel raises (3 × 15), plank (3 × 25 s), bicep curls (3 × 12)	2–3	Bodyweight exercise or moderate (~50–70% 1RM)	30
	Low-Level 4.3	Flexibility or Range of Motion	Stretches to improve leg mobility	2–3	Low	5
	Low-Level 4.4	Neuromotor Capabilities	Walking with arm swings	2–3	Low	
Visit 5 (Day 180 ± 15)	Low-Level 5.1	Cardiorespiratory Endurance	Walking at a brisk pace or stationary cycling	4–5	Moderate (~65–75% max HR)	30
	Low-Level 5.2	Muscle Strength and Endurance	Bodyweight squats (3 × 20), knee push-ups (3 × 12), lunges (3 × 20), heel raises (3 × 20), plank (3 × 30 s), bicep curls (3 × 15)	2–3	Bodyweight exercise or moderate (~50–70% 1RM)	35
	Low-Level 5.3	Flexibility or Range of Motion	Stretches for legs and back (low intensity)	2–3	Low	5
	Low-Level 5.4	Neuromotor Capabilities	Walking with changes in direction	2–3	Low	

1RM: one repetition maximum; HR: heart rate.

**Table 2:** Moderate-level physical exercise program: activities, frequency, intensity, and progression.

Visit	Level Exercise	Type of Exercise	Proposed Exercise	Frequency (Days/Week)	Intensity (~%HR max/~% 1RM)	Duration Per Type (min)
Visit 0 (Day 0)	Moderate-Level 1.1	Cardiorespiratory Endurance	Light jog or stationary bike	3–4	Moderate (~65–75% max HR)	25
	Moderate-Level 1.2	Muscle Strength and Endurance	Squats with light barbell (4 × 12), push-ups (3 × 10), lunges (3 × 10), heel raises (3 × 12), plank (3 × 15 sec), bicep curls (3 × 12)	2–3	Bodyweight exercise or moderate (~50–70% 1RM)	25
	Moderate-Level 1.3	Flexibility or Range of Motion	Low back and leg stretch	2–3	Low	10
	Moderate-Level 1.4	Neuromotor Capabilities	Walking on a straight line	2–3	Moderate	
Visit 2 (Day 30 ± 4)	Moderate-Level 2.1	Cardiorespiratory Endurance	Moderate jog or stationary bike at a constant pace	4–5	Moderate (~65–75% max HR)	30
	Moderate-Level 2.2	Muscle Strength and Endurance	Squats with moderate weight (4 × 12), push-ups (3 × 12), lunges (3 × 15), heel raises (3 × 15), plank (3 × 20 s), bicep curls (3 × 15)	2–3	Bodyweight exercise or moderate (~50–70% 1RM)	30
	Moderate-Level 2.3	Flexibility or Range of Motion	Trunk and hip stretches	3–4	Moderate	10
	Moderate-Level 2.4	Neuromotor Capabilities	Balance on one leg	2–3	Moderate	
Visit 3 (Day 60 ± 7)	Moderate-Level 3.1	Cardiorespiratory Endurance	Continuous jog or light intervals (walk/jog)	4–5	Moderate (~65–75% max HR)	35
	Moderate-Level 3.2	Muscle Strength and Endurance	Squats with barbell (4 × 12), push-ups (3 × 12), lunges (3 × 15), heel raises (3 × 15), plank (3 × 25 s), bicep curls (3 × 15)	2–3	Bodyweight exercise or moderate (~50–70% 1RM)	35
	Moderate-Level 3.3	Flexibility or Range of Motion	Advanced trunk stretches	3–4	Moderate	10
	Moderate-Level 3.4	Neuromotor Capabilities	Stair jumps	2–3	Moderate	
Visit 4 (Day 90 ± 7)	Moderate-Level 4.1	Cardiorespiratory Endurance	Light walk and jog intervals	5–6	Moderate (~65–75% max HR)	40
	Moderate-Level 4.2	Muscle Strength and Endurance	Squats with barbell (4 × 15), full push-ups (4 × 12), lunges (3 × 20), heel raises (3 × 20), plank (3 × 30 s), bicep curls (3 × 20)	3–4	Bodyweight exercise or moderate (~50–70% 1RM)	40
	Moderate-Level 4.3	Flexibility or Range of Motion	Low back and leg stretch	3–4	Low	10
	Moderate-Level 4.4	Neuromotor Capabilities	March with arm swings	2–3	Moderate	
Visit 5 (Day 180 ± 15)	Moderate-Level 5.1	Cardiorespiratory Endurance	Light walk and jog intervals	5–6	Moderate (~65–75% max HR)	45
	Moderate-Level 5.2	Muscle Strength and Endurance	Squats with barbell (4 × 12), push-ups (3 × 15), lunges (3 × 20), heel raises (3 × 20), plank (3 × 30 s), bicep curls (3 × 20)	3–4	Bodyweight exercise or moderate (~50–70% 1RM)	45
	Moderate-Level 5.3	Flexibility or Range of Motion	Deeper leg and low back stretches	3–4	Low	10
	Moderate-Level 5.4	Neuromotor Capabilities	Fast walking with direction changes	3–4	Moderate	

1RM: one repetition maximum; HR: heart rate.

**Table 3:** High-level physical exercise program: activities, frequency, intensity, and progression.

Visit	Level Exercise	Type of Exercise	Proposed Exercise	Frequency (Days/Week)	Intensity (~%HR max/~% 1RM)	Duration Per Type (min)
Visit 0 (Day 0)	High-Level 1.1	Cardiorespiratory Endurance	Moderate jog or intervals	5–6	Vigorous (~75–90% max HR)	35
	High-Level 1.2	Muscle Strength and Endurance	Squats with barbell (4 × 12), push-ups (4 × 12), lunges (4 × 15), heel raises (4 × 15), plank with lift (4 × 15 s), dumbbell rows (4 × 12)	3–4	Bodyweight exercise or vigorous (~70–85% 1RM)	40
	High-Level 1.3	Flexibility or Range of Motion	Deep stretches	2–3	Moderate	5
	High-Level 1.4	Neuromotor Capabilities	Advanced dynamic balance exercises	2–3	Vigorous	
Visit 2 (Day 30 ± 4)	High-Level 2.1	Cardiorespiratory Endurance	Jog or stationary bike at a steady pace	5–6	Vigorous (~75–90% max HR)	40
	High-Level 2.2	Muscle Strength and Endurance	Squats (4 × 12), push-ups (4 × 12), lunges (4 × 12), heel raises (4 × 12), plank (4 × 12 s), dumbbell rows (4 × 12)	3–4	Bodyweight exercise or vigorous (~70–85% 1RM)	45
	High-Level 2.3	Flexibility or Range of Motion	Dynamic stretches	2–3	Moderate	15
	High-Level 2.4	Neuromotor Capabilities	Complex coordination exercises (ladder, jumps)	2–3	Vigorous	
Visit 3 (Day 60 ± 7)	High-Level 3.1	Cardiorespiratory Endurance	High-intensity intervals (HIIT)	5–6	Vigorous (~75–90% max HR)	45
	High-Level 3.2	Muscle Strength and Endurance	Squats (4 × 12), push-ups (4 × 12), lunges (4 × 12), heel raises (4 × 12), plank with lift (4 × 15 s), dumbbell rows (4 × 12)	3–4	Bodyweight exercise or Vigorous (~70–85% 1RM)	50
	High-Level 3.3	Flexibility or Range of Motion	Advanced stretches	2–3	Moderate	15
	High-Level 3.4	Neuromotor Capabilities	Advanced coordination exercises (agility ladder)	2–3	Vigorous	
Visit 4 (Day 90 ± 7)	High-Level 4.1	Cardiorespiratory Endurance	HIIT (vigorous intensity intervals)	5–6	Vigorous (~75–90% max HR)	50
	High-Level 4.2	Muscle Strength and Endurance	Squats (4 × 12), advanced push-ups (4 × 12), lunges (4 × 12), heel raises (4 × 12), plank with lift (4 × 12 s), dumbbell rows (4 × 12)	3–4	Bodyweight exercise or vigorous (~70–85% 1RM)	55
	High-Level 4.3	Flexibility or Range of Motion	Dynamic stretches	2–3	Moderate	15
	High-Level 4.4	Neuromotor Capabilities	Advanced balance exercises with unstable platform	2–3	Vigorous	
Visit 5 (Day 180 ± 15)	High-Level 5.1	Cardiorespiratory Endurance	HIIT with combined strength work	5–6	Vigorous (~75–90% max HR)	55
	High-Level 5.2	Muscle Strength and Endurance	Squats (4 × 12), advanced push-ups (4 × 12), lunges (4 × 12), heel raises (4 × 12), plank (4 × 15 s), dumbbell rows (4 × 12)	3–4	Bodyweight exercise or vigorous (~70–85% 1RM)	60
	High-Level 5.3	Flexibility or Range of Motion	Deep stretches	2–3	Moderate	15
	High-Level 5.4	Neuromotor Capabilities	Advanced dynamic balance exercises	2–3	Vigorous	

1RM: one repetition maximum; HR: heart rate.

**Table 4:** Sequence of taking cytisinicline.

Days of Treatment	Recommended Dosage (1.5 mg)	Maximum Daily Dose
From the 1st to the 3rd day	1 tablet every 2 h	6 tablets
From the 4th to the 12th day	1 tablet every 2.5 h	5 tablets
From the 13th to the 16th day	1 tablet every 3 h	4 tablets
From the 17th to the 20th day	1 tablet every 5 h	3 tablets
From the 21st to the 25th day	1–2 tablets per day	2 tablets

## Data Availability

Data are contained within the article.
